# Enteral broad-spectrum antibiotics antagonize the effect of fecal microbiota transplantation in preterm pigs

**DOI:** 10.1080/19490976.2020.1849997

**Published:** 2020-12-31

**Authors:** Anders Brunse, Simone Margaard Offersen, Josefine Juliane Mosegaard, Ling Deng, Peter Damborg, Dennis Sandris Nielsen, Per Torp Sangild, Thomas Thymann, Duc Ninh Nguyen

**Affiliations:** a Section for Comparative Pediatrics and Nutrition, Department of Veterinary and Animal Sciences, Faculty of Health and Medical Sciences, University of Copenhagen, Copenhagen, Denmark; b Section of Food Microbiology and Fermentation, Department of Food Science, Faculty of Science, Copenhagen University, Copenhagen, Denmark; c Section for Veterinary Clinical Microbiology, Department of Veterinary and Animal Sciences, Faculty of Health and Medical Sciences, University of Copenhagen, Copenhagen, Denmark.

**Keywords:** Gut microbiota, antibiotics, fecal microbiota transplantation, prematurity, necrotizing enterocolitis, antibiotics resistance, immunity

## Abstract

Preterm infants are at risk of multiple morbidities including necrotizing enterocolitis (NEC). Suspected NEC patients receive intravenous antibiotics (AB) to prevent sepsis, although enteral AB is arguably more effective at reducing NEC but is rarely used due to the risk of AB resistance. Fecal microbiota transplantation (FMT) has shown protective effects against NEC in animal experiments, but the interaction between AB and FMT has not been investigated in neonates. We hypothesized that administration of enteral AB followed by rectal FMT would effectively prevent NEC with negligible changes in AB resistance and systemic immunity. Using preterm piglets, we examined host and gut microbiota responses to AB, FMT, or a sequential combination thereof, with emphasis on NEC development. In a saline-controlled experiment, preterm piglets (n = 67) received oro-gastric neomycin (50 mg/kg/d) and amoxicillin-clavulanate (50/12.5 mg/kg/d) (hereafter AB) for four days after cesarean delivery, and were subsequently given rectal FMT from healthy suckling piglet donors. Whereas AB protected the stomach and small intestine, and FMT primarily protected the colon, the sequential combination treatment surprisingly provided no NEC protection. Furthermore, minor changes in the gut microbiota composition were observed in response to either treatment, although AB treatment decreased species diversity and increased AB resistance among coliform bacteria and Enterococci, which were both partly reversed by FMT. Besides, enteral AB treatment suppressed cellular and functional systemic immune development, which was not prevented by subsequent FMT. We discovered an antagonistic relationship between enteral AB and FMT in terms of NEC development. The outcome may depend on choice of AB compounds, FMT composition, doses, treatment duration, and administration routes, but these results challenge the applicability of enteral AB and FMT in preterm infants.

## Introduction

The gastrointestinal tract is a prominent host–microbe interface, and microbial colonization of the immature gut of preterm infants (born before completed 37 weeks of gestation) represents a great health risk to this vulnerable population. Preterm infants endure a prolonged period of initial gut colonization by facultative anaerobic Bacilli and subsequently Gammaproteobacteria, before transitioning to an obligate anaerobic gut microbiota, reflecting that of healthy term infants.^1–3^ Harboring a dysbiotic gut microbiota at a critical time in gastrointestinal and immunological development may increase the risk of serious infections.

A significant morbidity of preterm infants is necrotizing enterocolitis (NEC), a life-threatening inflammatory bowel condition involving gut barrier disruption and mucosa bacterial invasion.^4^ Based on a recent meta-analysis of fecal 16S rRNA gene amplicon sequencing data, showing increased Proteobacteria and reduced Bacteroidetes abundance in NEC infants, a connection between delayed anaerobic colonization of the preterm gut and NEC development seems plausible.^5^ Moreover, gut microbiota instability, perceived as frequent transitions between well-defined dysbiotic clusters of single facultative anaerobic dominance, has been shown to precede NEC development.^6^

Preterm infants with suspected NEC routinely receive intravenous antibiotics (AB) to reduce the risk of systemic infection. However, enteral antibiotics (AB), specifically aminoglycosides, appear to reduce NEC incidence in preterm infants,^7^ which is in agreement with the effects seen in experimental NEC models,^8,9^ whereas prolonged intravenous AB is associated with an increased risk of NEC.^10,11^ On the other hand, studies in mice and chicken show increased AB resistance after enteral relative to injected ampicillin or tetracyclin administration,^12,13^ thereby limiting the use of enteral AB for NEC prophylaxis. Moreover, neonatal gut microbiota depletion by enteral AB treatment appears to hamper gut microbiota-mediated myelopoiesis and resistance to systemic infection.^14,15^

Fecal microbiota transplantation (FMT) refers to the transfer of fecal material containing microorganisms from donor to recipient with the intent of affecting the microbiota of the recipient.^16^ Using preterm pigs, we recently showed that early neonatal FMT shifted the gut microbiota toward anaerobicity, increased circulating leukocyte levels, and protected against NEC, but also involved severe tolerability issues if administered orally, emphasizing that its use can be a double-edged sword.^17^ Moreover, FMT accelerates immune reconstitution after AB-induced microbiota depletion.^18^ The only current clinical indication for FMT treatment is recurrent *Clostridioides difficile* infection where FMT, besides ensuring clinical remission, reduces the degree of AB resistance.^19^ Similarly, FMT treatment may promote immune maturation and reduce AB resistance in AB-treated preterm neonates.

The aim of this study was to combine enteral AB and rectal FMT, which both work well separately to prevent NEC, into a more clinically efficient treatment paradigm that reduce the unwanted effects of AB treatment such as AB resistance and arrested immunity development. We hypothesized that administration of enteral broad-spectrum AB followed by rectal FMT would effectively prevent NEC with negligible changes in antibiotic resistance and systemic immunity. To address this question, we treated preterm pigs with enteral antibiotics for the first four days of life followed by two days of rectal FMT and performed clinical, microbiological, and immunological evaluations on day 5 and 9.

## Results

### Enteral broad-spectrum antibiotics abolish the clinical effect of fecal microbiota transplantation

Resuscitation after preterm cesarean delivery was unsuccessful in nine of 76 animals, whereas the remaining ones were evenly allocated into four groups receiving enteral AB or control saline (CON) followed by rectal FMT or control saline (CON). Three allocated animals died early (excessive blood loss or respiratory failure), and another four were euthanized ahead of schedule and removed from the experiment due to iatrogenic complications. Among the remaining 60 animals, eight had to be euthanized preschedule [3 CON-CON (all NEC), 2 AB-CON (both NEC), 2 AB-FMT (both NEC), and 1 CON-FMT (sepsis/renal failure)] but were included in the analyses. Of these, none of the four AB-treated animals showed signs of NEC during the AB treatment phase, whereas in this period three CON-CON animals were euthanized and diagnosed with NEC.

**Figure 1.**
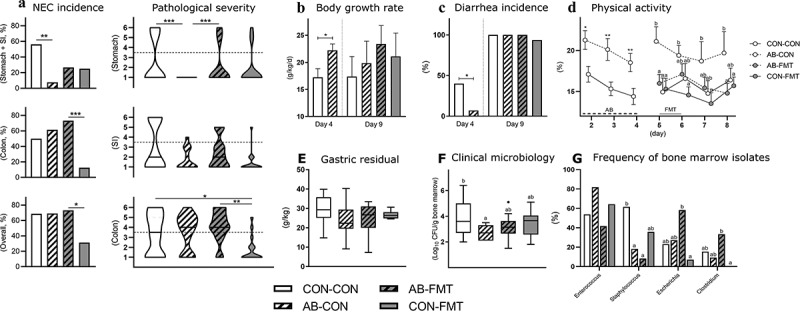
Enteral broad-spectrum antibiotics abolish the clinical effect of fecal microbiota transplantation. (a). NEC incidences (left panels) and lesion severities (right panels) in stomach, small intestine (SI) and colon by macroscopic pathological evaluation. Dotted horizontal lines specify the criteria for NEC diagnosis (score > 3). (b-d). Growth rate, diarrhea and in-cage physical activity. (e). Stomach emptying rate expressed as gastric residual volume after a timed standardized feeding bolus. (f-g). Bone marrow total bacterial density and frequencies of dominant isolates summarized at genus level. Ordinal data (NEC scores) is presented as violin plots with the solid horizontal line denoting the median value. Continuous data is presented as bar plots using means and standard error if normally distributed or otherwise using box plots with median and Tukey whiskers. n = 13–16 per group for all analyses. For two-group comparisons before day 5, *, ** and *** denote probability values of 0.05, 0.01 and 0.001. For four-group comparisons on day 9, data not sharing the same superscript letter are significantly different at *p* < .05

NEC incidence was markedly reduced in stomach and small intestine after AB treatment (CON-CON vs. AB-CON, *p* < .01, Figure 1a). Conversely, the colonic NEC incidence was lowest in FMT-treated animals without prior AB treatment (AB-FMT vs. CON-FMT, *p* < .001). Hence, the overall NEC incidence was reduced only in the CON-FMT group (*p* < .05 vs. AB-FMT). Accordingly, the pathological severity of the stomach was clearly reduced by AB treatment (CON-CON vs. AB-CON, *p* < .001), but the beneficial effect was completely abolished by subsequent FMT treatment (AB-CON vs. AB-FMT, *p* < .001). Whereas no differences were observed in the small intestine, the pathological severity in the colon was reduced by FMT (CON-CON vs. CON-FMT, *p* < .05), but only without prior AB treatment (AB-FMT vs. CON-FMT, *p* < .01).

In the continuous clinical monitoring data, we found beneficial effects during AB treatment that largely disappeared after AB discontinuation. Body growth rate was higher (*p* < .05, Figure 1b) and diarrhea incidence lower (*p* < .05, Figure 1c) after 4 days in AB animals but not different at day 9. Moreover, in-cage physical activity was higher in AB animals until AB discontinuation (all *p* < .05, Figure 1d), but after introduction of FMT, the physical activity in the AB-FMT group decreased relative to AB-CON on days 5 and 8 (both *p* < .05). Functional tests of gastric emptying (Figure 1e) and intestinal permeability (not shown) were not different among groups. However, AB-CON animals had lower numbers of cultivable bacteria in the bone marrow at euthanasia compared with CON-CON (*p* < .05, figure 1f). Of note, the AB-FMT group had significantly higher frequencies of Escherichia and Clostridia isolates in bone marrow than CON-FMT (both *p* < .05, Figure 1g).

### Minor effects of enteral broad-spectrum antibiotics and fecal microbiota transplantation on gut microbiota composition

**Figure 2.**
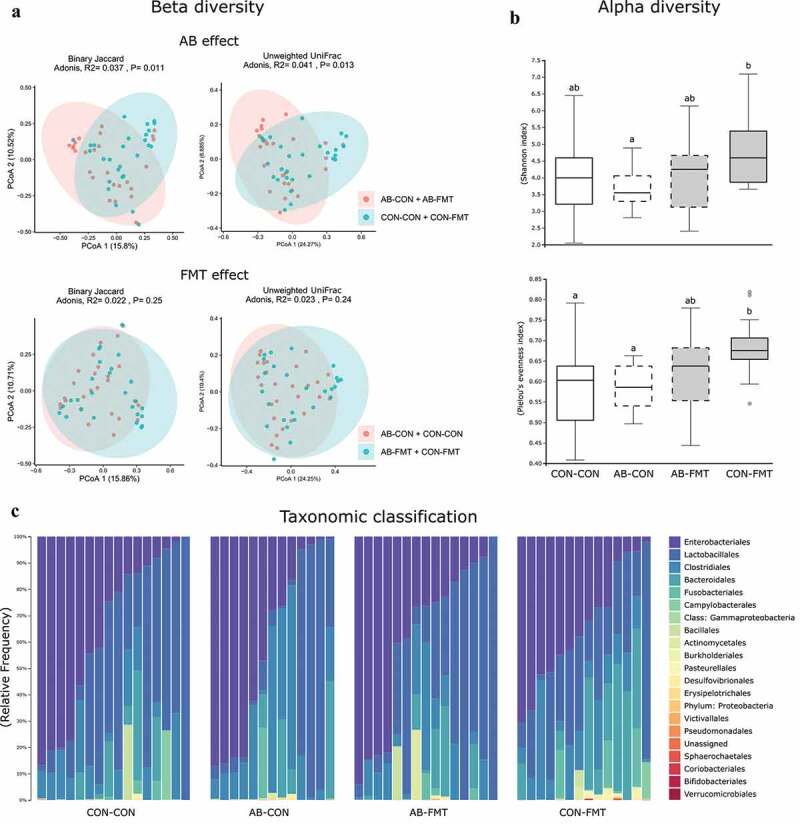
Minor effects of enteral broad-spectrum antibiotics and fecal microbiota transplantation on gut microbiota composition. (a). Dissimilarity plots based on 16S rRNA gene amplicon sequencing data, visualizing differences in beta diversity of the colonic microbiota after AB (upper) and FMT (lower) treatment using Jaccard (left) and Unweighted UniFrac distances (right). (b). Shannon and Pielou’s evenness indices as measures of alpha diversity (data not sharing the same superscript letter are significantly different at *p* < .05). (c). Relative colonic bacterial abundance presented as stacked bar graphs summarized at order level of taxonomic classification. n = 13–16 per group for all analyses

The colon bacterial microbiota in 9-day-old animals was marginally affected by AB treatment when assessed by qualitative measures of beta diversity, whereas no effect of FMT was observed (Figure 2a). Likewise, no difference in beta diversity was observed for any treatment in Bray-Curtis and weighted UniFrac dissimilarities (not shown). Alpha diversity tended to decrease after AB treatment and increase after FMT, when expressed as Shannon and Pielou’s evenness indices (AB-CON vs. CON-FMT, both *p* < .05, Figure 2b), whereas the number of observed species per sample was not different among the groups (not shown). Single taxa comparisons by ANCOM analysis yielded no significant differences at any taxonomic hierarchy from phylum to genus level (data summarized at order level, Figure 2c).

### Fecal microbiota transplantation reduces antibiotics-induced cefotaxime resistance

**Figure 3.**
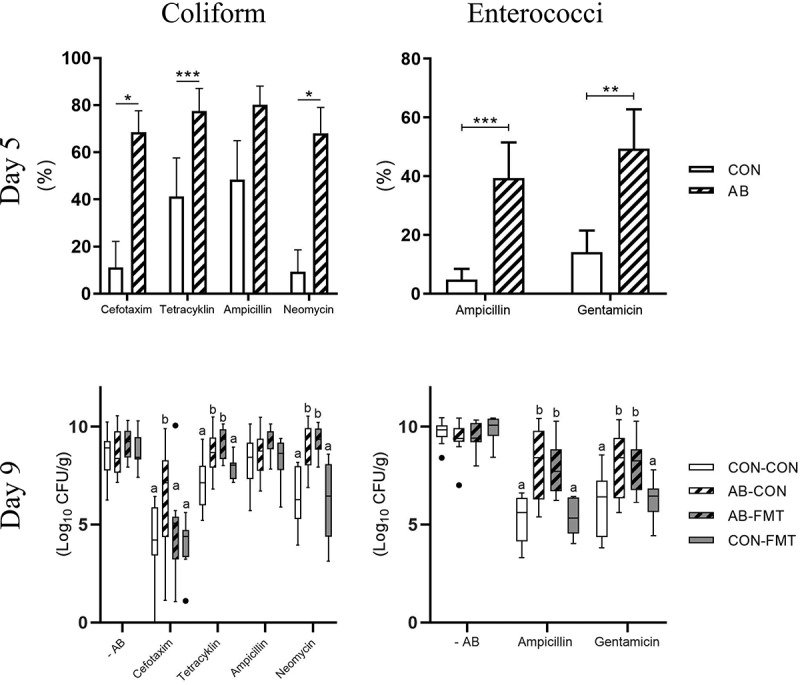
Fecal microbiota transplantation reduces antibiotics-induced cefotaxime resistance. Antibiotics resistant coliform bacteria (left, MacConkey agar) and enterococci (right, Slanetz-Bartley agar) on day 5, shortly after AB discontinuation (upper, presented as frequency of antibiotics resistant colony-forming units) and day 9, after subsequent FMT treatment (lower, presented as total number of antibiotics resistant colony-forming units). n = 9–16 per group. Continuous data are presented as bar plots using means and standard error if normally distributed or otherwise using box plots with median and Tukey whiskers. For two-group comparisons on day 5, *, ** and *** denote probability values of 0.05, 0.01 and 0.001. For four-group comparisons on day 9, data not sharing the same superscript letter are significantly different at *p* < .05

On day 5, less than 12 h after the final AB treatment, the median bacterial concentration of enema fluid was several orders of magnitude lower in AB animals relative to CON on MacConkey [12.5(IQR: 6.9) vs 2.0(3.3)*10 CFU/ml, *p* < .001 by Mann-Whitney U test] and Slanetz-Bartley agar [16.7(281) vs 3.9(1.9)*10 CFU/ml, *p* < .001]. Hence, only relative AB resistance was estimated in these samples. At this time, the relative proportions of coliform bacteria and enterococci displaying AB resistance were higher in AB-treated vs. CON animals for all tested AB compounds except ampicillin (Figure 3, upper panels).

Conversely, on day 9, five days after AB cessation, we found similar total CFUs of coliforms and enterococci across all four groups. Since Enterobacteriaceae and Enterococcaceae together constituted more than 50% of colonic bacterial abundance (Figure 2c), the absolute colonic bacterial load appeared similar among groups at this stage. Importantly, increased CFUs of cefotaxime-resistant coliforms after AB treatment decreased to control levels after FMT treatment (AB-CON vs. AB-FMT, *p* < .05, Figure 3, lower left panel). AB treatment also selected for tetracycline- and neomycin-resistant coliforms, but FMT had no modulating effect on these. Moreover, AB treatment led to increased CFUs of ampicillin- and gentamicin-resistant enterococci with no effect of subsequent FMT treatment (Figure 3, lower right panel). Expressing the day 9 data as relative AB resistance yielded similar results (data not shown).

### Enteral broad-spectrum antibiotics reduce small intestinal bacterial adhesion and mucosal immune cell densities

**Figure 4.**
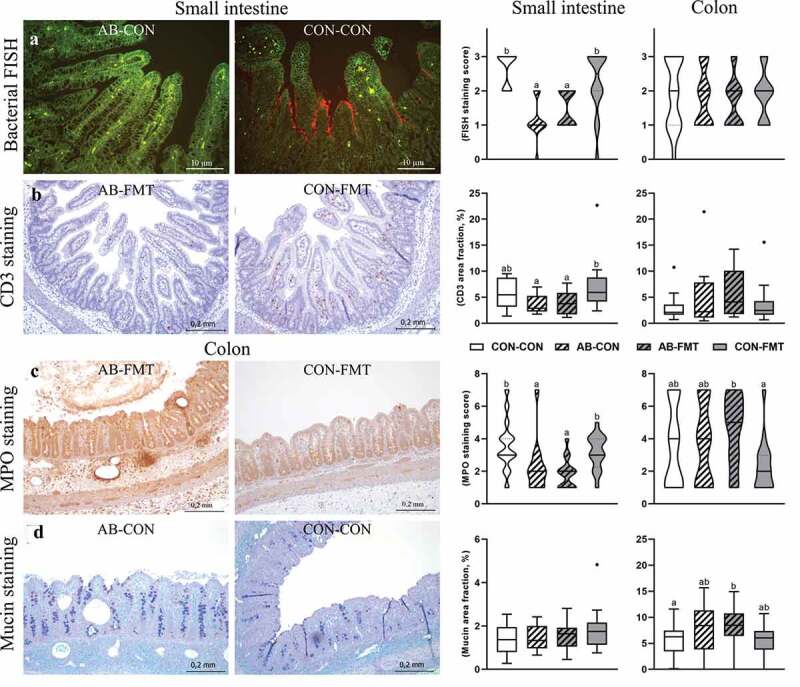
Enteral broad-spectrum antibiotics reduce small intestinal bacterial adhesion and mucosal immune cell densities. (a). Representative micrographs of AB-CON (left) and CON-CON (right) bacterial FISH stained small intestines. FISH stained tissue (small intestine and colon) was evaluated using an ordinal density grading system (score 0–3). (b). Representative micrographs of CD3 stained small intestines from AB-FMT (left) and CON-FMT (right) animals. The relative area of CD3 chromogenic signal in small intestines and colon was quantified by image analysis. (c). Representative micrographs of MPO stained colon from the AB-FMT group (left) and CON-FMT (right). Most AB-FMT tissue sections presented with MPO positive immune cell aggregates, pneumatosis intestinalis and submucosal thickening, whereas the majority of CON-FMT tissue sections had normal appearance. MPO stained tissue was evaluated using a composite ordinal grading system (0–7) consisting of MPO cell density (score 1–3) and severity/extent of MPO-associated mucosa inflammation (score 0–4). D. Representative micrographs of mucin stained colonic tissue using AB-PAS histochemistry showing AB-CON (left) and CON-CON (right) groups. The relative mucin stained area was quantified using image analysis. Ordinal data is presented as violin plots with median (solid line) and interquartile range (dotted line). Continuous data is presented as box plots showing medians and Tukey whiskers. n = 12–16 per group. Data not sharing the same letter are significantly different at *p* < .05. AB-PAS, Alcian Blue-Periodic acid-Schiff; MPO, myeloperoxidase; FISH, fluorescent *in situ* hybridization

Small intestinal tissue architecture was generally intact, whereas colon microstructure was often disrupted (e.g. by submucosal edema, blood congestion/enlarged vessels, immune cell aggregation, pneumatosis intestinalis, or ulceration). However, bacterial adhesion to the epithelium was most pronounced in the distal small intestine where we found a dramatic reduction after AB treatment (Figure 4a). Similarly, AB treatment decreased the CD3 stained area of the small intestine (as a measure of T cell density) relative to CON (*p* < .05 for AB-FMT vs CON-FMT, Figure 4b). Moreover, the MPO staining score of the small intestine (as a measure of macrophage and neutrophil granulocyte density) was significantly lower in both AB-treated groups compared with CON (both *p* < .05, Figure 4c), whereas FMT decreased the MPO score in the colon, but only without prior AB treatment (*p* < .05 for AB-FMT vs CON-FMT). No obvious differences in mucin density were observed, although densities in colon tended to be higher in AB-treated animals (Figure 4d).

A consistent pattern of concentration levels was found for the cytokines IL-6, CXCL-8 and TNF-α in colon tissue with highest levels in the CON-FMT group (all *p* < .05 vs. AB-FMT, Suppl. Fig. S1). Cytokine levels measured in small intestinal tissue homogenates (IL-1β, CXCL-8, IL-10, and TNF-α) were not different among groups (data not shown).

### Enteral broad-spectrum antibiotics attenuate blood lymphocyte dynamics

**Figure 5.**
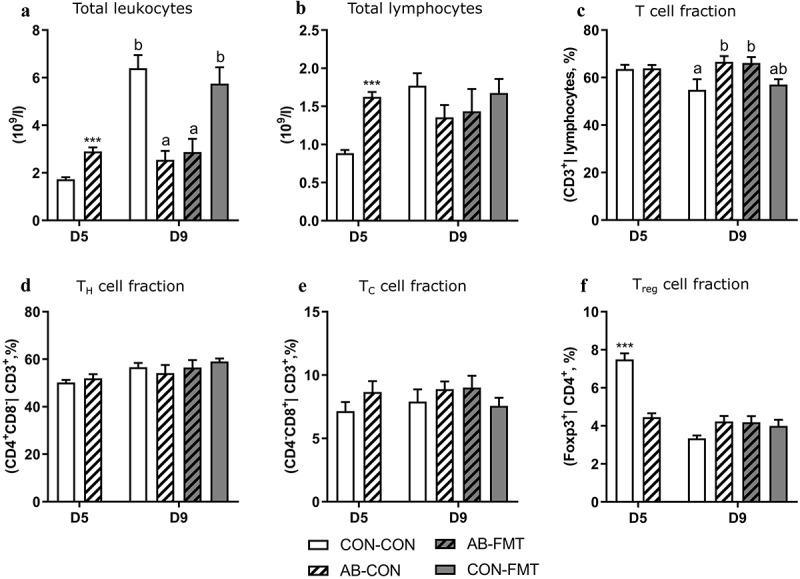
Enteral broad-spectrum antibiotics attenuate blood lymphocyte dynamics. (a-b). Total blood leukocyte and lymphocyte counts. (c-f). Frequency of T cells (CD3^+^ lymphocytes), T_H_ cells (CD3^+^CD4^+^CD8^−^ lymphocytes), T_C_ cells (CD3^+^CD4^−^CD8^+^ lymphocytes) and T_reg_ cells (CD3^+^CD4^+^Foxp3^+^ lymphocytes). Data are presented as means with standard errors. n = 6–16 per group. For two-group comparisons on day 5, *** denotes a probability value of 0.001. For four-group comparisons on day 9, data not sharing the same superscript letter are significantly different at *p* < .05

On day 5, shortly after AB discontinuation, numbers of circulating leukocytes as well as the lymphoid subset were substantially higher in AB animals compared with controls (both *p* < .001, Figure 5a-B). At this stage, no differences were observed for circulating T cell (CD3^+^ | FSC-SSC lymphocyte gate, Figure 5c), T_H_ (CD4^+^CD8^−^ | CD3^+^, Figure 5d) and T_C_ (CD4^+^CD8^−^ | CD3^+^, Figure 5e) cell frequencies, whereas the T_reg_ (Foxp3^+^ | CD3^+^ CD4^+^) cell frequency was significantly increased in CON relative to AB animals (*p* < .001, figure 5f). On day 9, five days after AB discontinuation, the trend in circulating leukocyte numbers was reversed and significantly lower in AB animals (*p* < .05, Figure 5a) due to a robust increase in CON-CON and CON-FMT from day 5 to 9 that did not occur in the two AB-treated groups. Circulating lymphocytes showed a similar trend albeit not reaching significantly higher levels in CON-CON and CON-FMT relative to AB-CON and AB-FMT (Figure 5b). On day 9, the T cell frequency was marginally increased in AB-CON relative to CON-CON (*p* < .05, Figure 5c), whereas none of the observed T cell subsets were different at this stage. No lymphocyte subsets were affected by FMT treatment.

### Enteral broad-spectrum antibiotics perturb blood myeloid cell composition and suppress immune function

**Figure 6.**
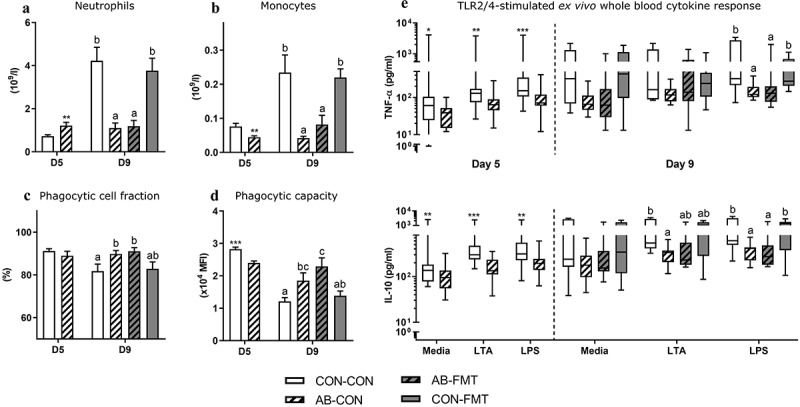
Enteral broad-spectrum antibiotics perturb blood myeloid cell composition and suppress immune function. (a-b). Total blood neutrophil and monocyte counts. (c-d). Neutrophil *ex vivo* phagocytic cell frequency and capacity. (e). TLR2 (LTA) and TLR4 (LPS) agonist stimulated cytokine secretion in *ex vivo* whole blood. Normally distributed data are presented as bar plots showing means with standard errors, whereas non-normally distributed data is presented as box plots with Tukey whiskers. n = 8–16 per group. For two-group comparisons on day 5, *, ** and *** denote probability values of 0.05, 0.01 and 0.001. For four-group comparisons on day 9, data not sharing the same superscript letter are significantly different at *p* < .05

On day 5, at the time of AB discontinuation, circulating neutrophil levels were higher in AB animals compared with CON (*p* < .01, Figure 6a). Four days later, in accordance with trends observed for blood lymphocytes, we recorded similar levels in AB animals, whereas CON animals showed a fivefold increase in neutrophil numbers compared with day 5. The expansion of circulating neutrophil numbers in CON relative to AB on day 9 was accompanied by a reduction in phagocytic cell fraction and phagocytic capacity, reflecting an immature functional state of these cells (AB-CON vs CON-CON, both *p* < .05, Figure 6c-d). In contrast, monocyte levels were elevated in CON relative to AB animals already at five days of age, and several folds higher on day 9 (all *p* < .05, Figure 6b). In an assessment of immune response capacity *ex vivo*, AB treatment attenuated TNF-α and IL-10 secretion relative to CON in both antigen-stimulated and non-stimulated whole blood on day 5 (all *p* < .05, Figure 6e). On day 9, TNF-α and IL-10 secretion was attenuated by AB relative to CON only in LPS-stimulated whole blood irrespective of subsequent FMT (all *p* < .05), while IL-10 was also attenuated by AB in response to LTA stimulation (AB-CON vs CON-CON, *p* < .05).

In addition, we found a uniform pattern of decreased immune-related gene expression in *ex vivo* LPS stimulated and unstimulated blood of AB-CON and AB-FMT relative to CON-CON and CON-FMT on day 9 (Table 1). Gene expression changes occurred for most assessed genes related with antigen recognition (*TLR2/4*), reactive immune response (e.g. *CXCL9*/*10, IFNG, IL12*/*17* as well as T_H_1/17 transcription factors *TBX21* and *RORC*, respectively), suppressive immune response (*IL10, TGFB1*) and glucose metabolism (*HK1, PKM, PDHA1*, and *HIF1A*). Collectively, major development occurred over time in circulating lymphoid and myeloid cellular composition and function in CON animals, whereas no developmental pattern was observed in animals receiving AB for the first four days of life. Conversely, FMT had no effect on systemic immunity, irrespectively of antecedent AB treatment.

**Table 1.** Gene expression profile in ex vivo LPS-stimulated whole blood

GENE	LPS	CON-CON		AB-CON		AB-FMT		CON-FMT	
*CXCL9*	-		1.00 ± 0.5^ab^		0.22 ± 0.1^ab^		0.08 ± 0.0^a^		1.52 ± 1.1^b^
	+		0.64 ± 0.2		0.19 ± 0.1		0.09 ± 0.0		0.32 ± 0.1
*CXCL10*	-		1.00 ± 0.5 ^c^		0.16 ± 0.1^ab^		0.09 ± 0.0^a^		0.71 ± 0.4^bc^
	+		0.73 ± 0.4^bc^		0.21 ± 0.1^ab^		0.17 ± 0.1^a^		0.57 ± 0.2 ^c^
*HIF1A*	-		1.00 ± 0.2		0.61 ± 0.2		0.64 ± 0.2		0.92 ± 0.2
	+		0.92 ± 0.1		0.65 ± 0.1		0.53 ± 0.1		0.89 ± 0.1
*HK1*	-		1.00 ± 0.2		0.69 ± 0.3		0.35 ± 0.1		0.75 ± 0.2
	+		1.56 ± 0.3 ^c^		0.77 ± 0.2^ab^		0.45 ± 0.1^a^		1.24 ± 0.2^bc^
*IFNG*	-		1.00 ± 0.6^ab^		0.15 ± 0.1^a^		0.08 ± 0.0^a^		1.61 ± 0.6^b^
	+		1.17 ± 0.9		0.17 ± 0.1		0.04 ± 0.0		0.38 ± 0.1
*IL10*	-		1.00 ± 0.4^ab^		0.25 ± 0.2^a^		0.17 ± 0.1^ab^		0.92 ± 0.2^b^
	+		1.12 ± 0.3^bc^		0.36 ± 0.1^ab^		0.25 ± 0.1^a^		1.14 ± 0.1 ^c^
*IL12*	-		1.00 ± 0.3		0.33 ± 0.2		0.47 ± 0.2		0.89 ± 0.4
	+		1.07 ± 0.3^ab^		0.30 ± 0.1^ab^		0.42 ± 0.2^a^		1.76 ± 0.5^b^
*IL17*	-		1.00 ± 0.4^b^		0.22 ± 0.1^a^		0.24 ± 0.1^a^		0.57 ± 0.3^ab^
	+		1.04 ± 0.4^ab^		0.11 ± 0.1^a^		0.28 ± 0.2^a^		1.13 ± 0.4^b^
*PDHA1*	-		1.00 ± 0.1		0.81 ± 0.2		0.82 ± 0.1		0.99 ± 0.2
	+		1.05 ± 0.2^ab^		0.72 ± 0.1^a^		0.80 ± 0.1^a^		1.45 ± 0.2^b^
*PKM*	-		1.00 ± 0.2		0.73 ± 0.2		0.83 ± 0.3		1.04 ± 0.3
	+		1.20 ± 0.2^b^		0.63 ± 0.1^a^		0.63 ± 0.1^a^		1.29 ± 0.2^b^
*RORC*	-		1.00 ± 0.2^ab^		0.54 ± 0.2^ab^		0.43 ± 0.1^a^		1.26 ± 0.4^b^
	+		1.10 ± 0.2^bc^		0.40 ± 0.1^a^		0.45 ± 0.1^ab^		1.61 ± 0.5 ^c^
*PPARA*	-		1.00 ± 0.2^b^		0.75 ± 0.1^ab^		1.15 ± 0.2^b^		0.53 ± 0.1^a^
	+		0.79 ± 0.1		0.75 ± 0.1		0.79 ± 0.1		0.81 ± 0.1
*TBX21*	-		1.00 ± 0.3^b^		0.46 ± 0.1^a^		1.05 ± 0.6^ab^		1.03 ± 0.3^ab^
	+		1.45 ± 0.5^bc^		0.67 ± 0.1^ab^		0.58 ± 0.2^a^		1.21 ± 0.3 ^c^
*TGFB1*	-		1.00 ± 0.1^b^		0.76 ± 0.2^a^		0.77 ± 0.1^ab^		0.76 ± 0.1^ab^
	+		0.83 ± 0.1^ab^		0.68 ± 0.1^ab^		0.59 ± 0.1^a^		1.06 ± 0.2^b^
*TLR2*	-		1.00 ± 0.3^ab^		0.46 ± 0.2^a^		0.40 ± 0.1^a^		1.50 ± 0.3^b^
	+		1.80 ± 0.3^ab^		0.99 ± 0.3^a^		0.76 ± 0.1^a^		2.32 ± 0.3^b^
*TLR4*	-		1.00 ± 0.3^ab^		0.42 ± 0.2^a^		0.25 ± 0.1^a^		1.56 ± 0.3^b^
	+		1.95 ± 0.4^b^		0.65 ± 0.2^a^		0.52 ± 0.2^a^		1.87 ± 0.1^b^

Target genes are ordered alphabetically according to gene name. Only genes with significant group effects are shown. Data is presented as means with standard errors. For each primer assay, the mean expression level of the control group (CON-CON) has been normalized to 1 and all values scaled accordingly. Group values in each row not sharing the same superscript letter are significantly different at a probability of 0.05 or lower.

## Discussion

Contrary to our a priori assumption, the sequential combination therapy of enteral neomycin and amoxicillin-clavulanate treatment with FMT was clinically inferior to both monotherapies in terms of NEC development. The experimental design allowed us to evaluate both isolated and combined treatment effects. We initiated AB treatment shortly after preterm birth, and the isolated clinical effect of AB was characterized by a pronounced NEC protection in stomach and small intestine, whereas the colon was just as susceptible as in controls. Importantly, onset of clinical signs never occurred until after AB discontinuation, while signs of NEC appeared earlier in control animals. Accordingly, AB animals had a higher growth rate and lower diarrhea incidence during AB treatment. Conversely, the isolated effects of FMT, administered rectally on days 5–6 mainly prevented NEC in the colon.

As such, it would seem plausible that the sequential combination of enteral neomycin and amoxicillin-clavulanate with rectal FMT might act synergistically to prevent lesions along the entire gut. Instead, the group of animals receiving sequential AB and FMT treatment had a NEC incidence and lesion severity in each of the gastrointestinal regions similar to untreated controls, suggesting an antagonistic effect of enteral broad-spectrum AB on subsequent FMT treatment. Interestingly, we found consistently lower colonic cytokine levels in AB-FMT compared with CON-FMT, possibly indicating AB-induced hyporesponsiveness toward FMT bacteria. Furthermore, we found a much higher number of AB-FMT animals colonized with *Escherichia coli* and *Clostridium perfringens* in the bone marrow relative to CON-FMT. Possibly, the low gut bacterial density and hyporesponsive mucosal immune cells following AB treatment allow subsequent FMT-derived opportunistic pathogenic bacteria to occupy mucosal niches that would otherwise be inaccessible due to host defense mechanisms and the presence of commensal bacteria. We did not measure stool AB concentrations and hence cannot exclude that residual AB has affected the subsequent FMT response.

Clinical epidemiological data shows that prolonged intravenous AB exposure is associated with an increased risk of NEC in preterm infants,^10,11,20^ whereas short-duration early postnatal AB treatment is associated with a decreased risk.^21^ However, these studies could be biased by unrecognized co-morbidities and should be interpreted with caution. Placebo-controlled trials (7–24 days) of early-initiated enteral aminoglycosides performed in preterm infants collectively indicate a beneficial effect on NEC occurrence,^7^ primarily driven by a single trial showing a robust protective effect of 7 days of early enteral vancomycin treatment.^22^ In experimental models, the effect of enteral broad-spectrum AB is striking with complete protection against NEC development.^8,9^ However, these studies do not monitor clinical response, and microbiota and immune development beyond AB discontinuation. We previously reported no adverse clinical effects six days after discontinuation of a 5-day course of combined enterally and intravenously administered gentamicin, ampicillin, and metronidazole in a single litter of preterm formula-fed pigs,^23^ which is in sharp contrast to the current unequivocal finding of high colonic NEC incidence after AB discontinuation across three pig litters. This inconsistency could be due to differences in study design including AB compounds used, administration route or speed of enteral feeding advancement.^23^ As such, enteral broad-spectrum AB appears beneficial against NEC lesions of the stomach and small intestines, but discontinuation is associated with substantial colonic pathology.

The beneficial effect of FMT is a confirmation of a previous observation of ours, wherein preterm pigs received rectal FMT shortly after birth, which led to a substantially reduced risk of NEC.^17^ The donor fecal material used in the current experiment originates from the same batch that we used in the previous report. However, the NEC incidence in the current experiment was 31% relative to 12% previous achieved, and the relative risk reduction of 54% relative to 75% in the previous experiment. The most likely explanation for this difference in efficacy is the delayed time of FMT treatment initiation in the current study (day five vs day one), which implies that FMT is less likely to alleviate developing NEC relative to preventing it from birth before disease onset. Moreover, the timing of FMT treatment may affect the engraftment potential of the FMT donor bacteria, which may in turn affect the clinical outcome. We previously observed robust changes in the gut microbiota composition of preterm pigs receiving FMT on the day of birth including enrichment of *Lactobacillus* and *Bacteroides* that were likely donor-engrafted.^17^ In the current study, we found that FMT had much less impact on the gut microbiota composition, when administered on day 5, either with or without antecedent neomycin and amoxicillin-clavulanate. The relative abundance of *Lactobacillus* and *Bacteroides* in FMT recipients were lower than previously reported and not different from controls. In fact, no single bacterial genus was significantly different between any of the four groups assessed.

At the time of euthanasia (day 9), the animals had almost reached the same age as their FMT donor (10 days of age). Regardless, their gut microbiota composition was profoundly different. Whereas the donor microbiota was abundant in several anaerobic taxa and *Lactobacillus* as the major facultative genus,^17^ the majority of preterm pigs, irrespective of treatment group, had a facultative dysbiosis with dominance of either Lactobacillales (*Enterococcus*) or Enterobacteriales. Arguably, in this setting, the natural transition from facultative to obligate anaerobicity is constrained by factors such as host prematurity, cesarean delivery, artificial rearing, formula feeding, or a combination thereof. Collectively, this might explain why the effects of enteral neomycin and amoxicillin-clavulanate as well as FMT on the gut microbiota were relatively minor.

Epithelial oxygen retention is a key factor for gut colonization by anaerobic bacteria, and is inhibited by AB treatment, thereby increasing luminal oxygen concentration.^24^ This may lead to resistance to anaerobic colonization, delaying the natural gut microbiota development and in turn create a favorable niche for opportunistic facultatives.^25^ Indeed, although the utilized donor feces were overall beneficial, we recently identified invasive pathogenic *Escherichia coli* in this material (unpublished data). In a setting of low gut bacterial density and high luminal oxygen pressure after enteral broad-spectrum AB treatment, FMT-derived anaerobes might fail to engraft, whereas pathogenic *E. coli* could thrive. A study in newborn mice likewise indicated that in a setting of maternal gentamicin-induced gut dysbiosis, FMT failed to promote anaerobic colonization and epithelial oxygen retention.^26^ Hence, age might determine to what extent FMT engrafts its recipient, as an antecedent cocktail of neomycin, vancomycin and ampicillin appears to facilitate FMT engraftment in older individuals.^27^ Moreover, autologous FMT is superior to spontaneous recolonization and probiotics at reconstituting ciprofloxacin/metronidazole-induced dysbiosis in human adults,^28^ but autologous FMT is unlikely to be as effective in preterm infants due to their dynamic gut microbiota.

We previously observed that the gut microbiota in AB-treated preterm pigs (ampicillin, gentamicin, metronidazole cocktail) was completely dominated by ampicillin/gentamicin resistant Enterobacteriaceae.^9^ The current data then indicate that Enterococci quickly recolonizes the colon following AB discontinuation. On the other hand, we found increases in AB resistant coliforms and Enterococci four days after AB treatment discontinuation. Hence, whereas the gut microbiota composition appears dynamic, it maintains its antibiotic resistance capacity, at least short term. We found resistance toward ampicillin across all groups, but apart from that, AB treatment was selected for resistance toward a spectrum of AB compounds, including drugs not used in the treatment cocktail. Most importantly, AB treatment increased the abundance of cefotaxime resistance among coliforms (mainly *Enterobacter cloacae* and *Pseudomonas aeruginosa*), which is a feature of multi-drug resistant organisms. These bacteria are clinically challenging to manage and pose a serious threat to compromised patients such as preterm infants.^29^ Interestingly, FMT treatment reduced levels of cefotaxime resistance after AB treatment, which warrants further investigation. On the other hand, FMT is also capable of transmitting AB resistance genes to recipients, and likely to a greater degree if the recipient microbiota is naïve or dysbiotic, emphasizing the need for rigorous screening of donor stool.^30^

The distinct patterns of gross pathology between small intestine and colon among the treatment groups were recapitulated on the microscopic tissue level. We performed a series of basic stainings to assess epithelial barrier function and immune cell distribution. Principally, enteral neomycin and amoxicillin-clavulanate treatment markedly reduced the extent of epithelial bacteria adhesion in the small intestine, which was in line with a lower overall density of cultivable bacteria in the bone marrow, reflecting gut translocation in the first days after preterm birth. This was accompanied by lower small intestinal myeloid cell density. However, across all groups both T cells and myeloid cells were evenly scattered throughout a structurally intact small intestinal mucosa, suggesting that the increased cell density in animals not treated with AB was due to microbiota-dependent immune maturation and not *bona fide* inflammation. Conversely, colonic myeloid cell staining showed very low MPO expression (and largely intact tissue structure) only in the CON-FMT group, whereas the remaining groups had MPO-positive inflammatory foci in mucosa and submucosa, often in association with intramural gas pockets. As such, MPO-staining was the only histological measure that was well correlated with gross pathological NEC assessment.

Gut colonization influences not only mucosal immune cell compartments but also hematopoiesis,^14,15^ and consequently, enteral broad-spectrum AB use in the neonatal period has a large impact on developing immunity, raising concerns about both short- and long-term issues of neonatal AB exposure. In this experiment, we performed blood lymphocyte profiling and assessed blood myeloid cell function at the time of AB discontinuation and again at euthanasia. Five days after the final AB treatment, no numerical development had occurred in the circulating leukocyte pool. Moreover, AB-treated animals displayed systemic immune suppression, shown by diminished gene response and cytokine secretion following bacterial antigen challenge. This indicates the existence of a significant lag phase between gut recolonization following early enteral broad-spectrum AB treatment and subsequent immune cell expansion, and could be a window of heightened infection risk. Conversely, control animals had a transient decrease in circulating lymphocytes and neutrophils on day 5, possibly due to gut homing in response to colonization, both of which had replenished and expanded in numbers on day 9. Moreover, the phagocytic capacity of neutrophils in control animals was decreased at the late stage, indicating myelopoietic activity and recruitment of newly differentiated cells. NEC status was not associated with changes in systemic immune parameters.

While we did not challenge the immune capacity of these animals, we postulate that newborns treated with enteral broad-spectrum AB are less competent at clearing a secondary systemic bacterial infection for a certain period after AB discontinuation. Similarly, newborn mice with AB-induced myelopenia are more susceptible to neonatal sepsis.^14,15^ Moreover, septic preterm infants but not matched controls have a facultative dysbiosis closely resembling that of the preterm pig,^31,32^ collectively linking enteral AB-induced gut dysbiosis and arrested immune development with increased risk of neonatal sepsis. Of note, in this experiment, FMT did not affect any systemic immune parameters, which we had anticipated based on observations of increased blood lymphocytes and neutrophils in our previous FMT experiment as well as reports of accelerated immune reconstitution by FMT following AB-induced decolonization in older individuals.^17,18^

Unlike previous reports in adult individuals, reconstituting the gut microbiota using FMT after enteral broad-spectrum AB treatment, we show that this procedure is detrimental to preterm pigs. As this is the first study to examine this interaction in neonates, we still need to uncover how changes to important factors such as AB compounds, FMT donor bacteria (e.g. selecting beneficial consortia), dosing, timing, duration, and routes of administration may affect the integrated response. If properly optimized, one might turn the antagonistic relationship between AB and FMT into a synergistic one.

## Materials and methods

### Birth, resuscitation and group allocation

Animal experimental procedures were approved by the Danish Animal Experiments Inspectorate (license number, 2014–15-0201-00418), which is in accordance with the guidelines from Directive 2010/63/EU of the European Parliament. Seventy-six crossbred piglets [(Landrace x Large white) x Duroc mixed donor semen] were delivered by cesarean section at ~90% gestation (day 106 ± 1) from three healthy sows. Briefly, sows were premedicated (1 mg/kg tiletamin, 1 mg/kg zolazepam, 0.4 mg/kg butorphanol) and surgical anesthesia was induced and maintained with propofol and 2% isoflurane inhalation. A uterine incision was made, and fetuses removed after ligating and transecting the umbilical cords. Once delivered, the newborn animals were housed in ventilated, preheated (37°C) individual incubators with initial oxygen supply (1–2 l/min). For resuscitation, animals received Doxapram (0.1 ml/kg im.), Flumazenil (0.1 ml/kg im.), physical stimulation and positive airway pressure ventilation until respiration had stabilized. Animals were then fitted with umbilical (4 Fr gauge) and orogastric (6 Fr gauge) catheters, and administered an 8 h continuous intra-arterial infusion of 16 ml/kg sow plasma to compensate for the lack of transplacental immunoglobulin transfer. Pigs were stratified according to gender and birth weight and randomly allocated to four groups (CON-CON, AB-CON, AB-FMT and CON-FMT), initially receiving antibiotic treatment (AB) or control treatment (CON), followed by FMT or control treatment (CON) in a 2 x 2-factorial experiment.

### Feeding, antibiotics and fecal microbiota transplantation

Animals received infant formula by oro-gastric tube feeding every 3 hours in gradually increasing amounts from day 1–9 (16–112 ml/kg/d) with decreasing parenteral nutrition supplement [144–48 ml/kg/d Kabiven (Fresenius Kabi)]. Infant formula composition is presented in Supplementary table S1. Animals in the AB groups (AB-CON and AB-FMT) received an enteral AB cocktail twice daily for the first four days of life. The cocktail consisted of 50/12.5 mg/kg/d amoxicillin and clavulanic acid and 50 mg/kg/d neomycin. Control animals (CON-CON and CON-FMT) received water instead of the AB cocktail.

Donor fecal material was collected, handled, and prepared as previously described, and derived from the same batch that had shown beneficial effects against NEC.^17^ Before use, the frozen donor feces was thawed and diluted in sterile saline to working concentration (50 mg raw colon content per ml). Animals in the FMT groups (AB-FMT and CON-FMT) received a rectal inoculation of 0.5 ml FMT working solution twice daily on days 5 and 6 by inserting a soft cannula (6 Fr gauge) 5 cm into the rectum. Control animals (AB-CON and CON-CON) were subjected to the same procedure with sterile saline. The washout period between the final antibiotic treatment and the first FMT was exactly 12 h.

### Clinical assessment and in vivo procedures

Experienced personnel performed frequent clinical assessments throughout the study and euthanized any animal that would present with clinical signs of NEC or systemic illness (bloated abdomen, discoloration, circulatory and respiratory failure, unconsciousness). Stool characteristics (consistency and volume) were assessed daily, and diarrhea incidence reported. In-cage physical activity was measured with continuous infrared video surveillance connected to a motion detection software (PigLWin, Ellegaard Systems).^33^

On day 5, between the last AB treatment and the first FMT, animals were given an enema (3 ml/kg sterile saline) to recover a fraction of rectal content for AB resistance analysis. On day 5 and 9, arterial blood was collected from the umbilical catheter for immunity assays. On day 9, 3 hours before scheduled euthanasia, animals received 15 ml/kg of a 5/5% w/v lactulose and mannitol solution in the oro-gastric catheter to measure intestinal permeability, and 1 hour before euthanasia, animals were given a 15 ml/kg infant formula bolus to assess gastric emptying rate.

### Euthanasia, necropsy and tissue collection

On day nine, following four days of AB, two days of FMT and a three-day follow-up, animals were deeply anesthetized and euthanized with an intracardiac injection of barbiturate. After abdominal incision, urine was collected by bladder puncture for lactulose and mannitol measurement as previously described.^34^ Abdominal organs were excised and weighed including stomach residual content. Pathological changes to the stomach, small intestine, and colon were assessed in accordance with an established six-grade NEC scoring system.^17^ NEC was defined as pathology grade 4 (extensive hemorrhage) in at least one gastrointestinal segment, and both incidence and severity were reported. Colon content was collected and immediately subjected to phenotypic antibiotic resistance analysis or snap-frozen for gut microbiota analysis. Small intestinal and colonic tissues were snap-frozen and immersion fixed in 4% paraformaldehyde for later analyses. Finally, a bone marrow biopsy was collected for clinical microbiology, after dissecting and aseptically opening the distal epiphysis of the left femur. Bone marrow biopsies were homogenized in sterile saline, serially diluted, seeded onto tryptic soy agar with 5% calf blood, and incubated under aerobic conditions for 24 hours. Afterward, colonies were enumerated, subcultured, and isolates identified to species level by matrix-assisted laser desorption/ionization time-of-flight (Maldi-TOF) mass spectrometry (Vitek MS RUO, bioMerieux) using Escherichia coli ATCC 8739 as reference strain and the software Saramis TM 3.5 (bioMerieux) for spectra interpretation.

### Gut microbiota composition

Gut microbiota composition was determined by 16S rRNA gene (V3-region) amplicon high throughput NextSeq PE150 amplicon sequencing (Illumina). Total cellular DNA from colon content collected at euthanasia was extracted using Bead-Beat Micro AX Gravity Kit (A&A Biotechnology,) according to the manufacturer’s instructions. Library preparation followed a previously published protocol.^35^ For bioinformatics processing, the raw sequencing reads were merged and trimmed. Chimeras were removed and zero-radius Operational Taxonomic Units (zOTUs) constructed using UNOISE algorithm implemented in Vsearch.^36–38^ Greengenes (version 13.8) database was used as reference for annotation. Qiime2 (2019.04) was used to process the forward analysis. Rare zOTUs with frequency below 0.1% of the minimal sample depth were filtered and removed, and the zOTU table was rarified to adequate sample depth (4500 counts) for alpha and beta diversity calculation based on rarefaction curve. Principal coordinate analysis (PCoA) was conducted on Jaccard, Bray-Curtis, unweighted and weighted UniFrac distances, a PERMANOVA test was performed to detect statistical difference between groups, and probability values were adjusted after pairwise testing. Specific taxa comparison among groups was analyzed by ANCOM with default qiime2 settings used to test statistically significant difference.

### Antibiotic resistance

Day-5 enema fluid and day-9 colon content were serially diluted in saline, and 20 µl triplicates seeded onto MacConkey agar (coliforms) supplemented with 1 µg/ml cefotaxime, 16 µg/ml tetracycline, 32 µg/ml ampicillin or 16 µg/ml neomycin, and onto Slanetz-Bartley agar (enterococci) with 16 µg/ml ampicillin or 128 µg/ml gentamicin. The same agar plates without antibiotics served as normalization factor in day-5 enema fluid, and as reference in day-9 colon content. MacConkey agar plates were incubated for 24 h at 37°C, and Slanetz-Bartley cultures for 48 h at 42°C. Total colonies were enumerated, and dominant morphotypes were sub-cultured and identified by Maldi-TOF as described above.

### Gut histological analyses and cytokine measurements

Duplicate distal small intestinal and colonic tissue biopsies were embedded in paraffin, and four serial 5 µm sections were used for bacterial visualization by fluorescence in situ hybridization,^9^ mucin visualization by Alcian Blue-Periodic acid-Schiff staining,^17^ and T cell (anti-porcine CD3, clone 4510–01, Southern Biotech) and neutrophil/macrophage immune staining (anti-human MPO, clone A0398, Agilent). Anti-CD3 stained area was quantified by image analysis using the color deconvolution function in the ImageJ software package (National Institutes of Health,), whereas anti-MPO-stained sections were graded according to cellular density and degree of tissue inflammation by a blinded investigator. Cytokine (IL-1β, IL-6, CXCL-8, IL-10, TNF-α) concentrations in distal small intestinal and colonic tissue homogenates were measured using porcine specific DuoSet enzyme-linked immunosorbent assays (R&D Systems).

### Systemic immune cell profiling and functional assays

Blood samples collected on day 5 (after the last AB treatment) and day 9 (euthanasia) were subjected to routine hematology analysis (Advia 2120i Hematology System, Siemens), T cell profiling and neutrophil phagocytosis by flow cytometry, and toll-like receptor (TLR) stimulated immune response assay. T cell profiling and neutrophil phagocytosis assays were performed as previously described using similar antibodies, reagents, and equipment.^39^ T cell subsets were identified as helper T cells (T_H_, CD3^+^CD4^+^CD8^−^ lymphocytes), cytotoxic T cells (T_C_, CD3^+^CD4^−^CD8^+^ lymphocytes) and regulatory T cells (T_reg_, CD3^+^CD4^+^Foxp3^+^ lymphocytes). In the TLR stimulation assay, fresh whole blood was treated with 1 µg/mL lipopolysaccharide (LPS-EB, from *E.coli* O111:B4, Invivogen) or 5 µg/mL lipoteichoic acid (LTA-SA, from *S. aureus*, Invivogen) for 5 h at 37°C and 5% CO_2_. Stimulated blood was split into two aliquots and either centrifuged (2000 x *g*, 10 min, 4°C) and supernatant collected for ELISA measurements of TNF-α (DY690B, R&D Systems) and IL-10 (DY693B, R&D Systems) protein levels, or mixed with MagMAX lysis/binding solution (Thermo Fisher) and RNA extracted, for gene expression analysis as previously described.^40^ Relative mRNA expression of target genes was normalized to the expression level of the reference gene *HPRT1*. All primers were designed using Primer-Blast (National Institutes of Health) with sequences listed in Supplemental Table S2.

### Statistics

Categorical data (NEC, diarrhea, and bone marrow colonization incidence) were analyzed by Fisher’s exact test. Ordinal data (NEC severity, and MPO and bacterial FISH staining) were analyzed using ordered logistic regression (R-studio’s polr package) with Tukey’s posthoc testing of pairwise differences. Remaining continuous data were analyzed using a linear group-effects model with Tukey’s posthoc testing of pairwise differences between the four groups. All models were adjusted for litter, gender, and birth weight. For continuous datasets, non-normally distributed data were log-transformed to fit model assumptions. If this was not achieved, a Kruskal-Wallis test with Dunn’s post hoc testing was used instead. All statistical analyses were performed in R-studio v1.1.456. Test probability values below 0.05 were considered significant.

## Supplementary Material

Supplemental Material
