# Supplementary material for: Enteral broad-spectrum antibiotics antagonize the effect of fecal microbiota transplantation in preterm pigs
Source: Gut Microbes. 2020 Dec 31;13(1):1849997. doi: 10.1080/19490976.2020.1849997 (PMC7781584; doi:10.1080/19490976.2020.1849997)
Supplement: Supplemental Material [file KGMI_A_1849997_SM5658.docx]

**Supplementary material**

**Supplementary Figure S1***. Colonic cytokine levels.*


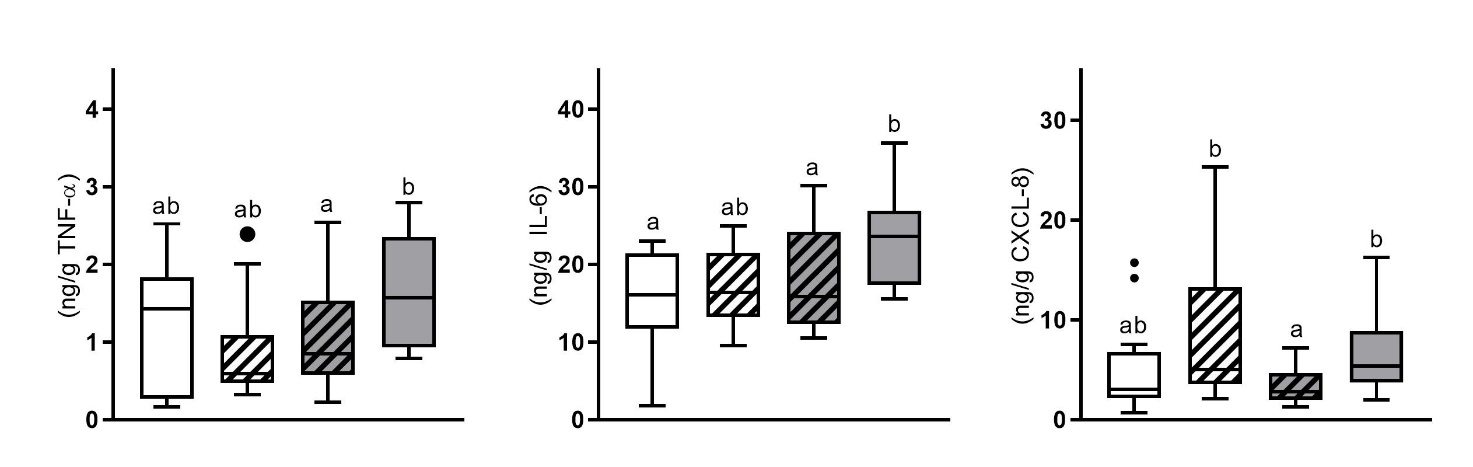


**Supplementary table S1**. *Infant formula composition*.

| Product | Amount (g/l) | Company |
| --- | --- | --- |
| SHS seravit, vitamin and mineral mix | 12 | Nutricia, Allerød, Denmark |
| SHS Liquigen MCT, lipid emulsion | 60 | Nutricia |
| Calogen LCT, lipid emulsion | 40 | Nutricia |
| Fantomalt, maltodextrin | 30 | Nutricia |
| Variolac, lactose powder | 10 | Arla Foods Ingredients, Viby, Denmark |
| Miprodan 40, casein | 35 | Arla Foods Ingredients |
| Lacprodan DI-9224, whey protein | 30 | Arla Foods Ingredients |
|  | | |

| **Symbol** | **Gene name** | **5’ primer sequence** | **3’ primer sequence** | **AL** |
| --- | --- | --- | --- | --- |
| *CXCL9* | Chemokine (C-X-C motif) ligand 9 | GAAAAGCAGTGTTGCCTTGCT | TGATGCAGGAACAACGTCCAT | 98 |
| *CXCL10* | Chemokine (C-X-C motif) ligand 10 | ATCATCCCGAGCTGTTGAGC | CCAGGACTTGGCACATTCAC | 94 |
| *GATA3* | GATA3 | ACCCCTTATTAAGCCCAAGC | TCCAGAGAGTCGTCGTTGTG | 92 |
| *HIF1A* | Hypoxia-inducible factor 1-alpha | TGTGTTATCTGTCGCTTTGAGTC | TTTCGCTTTCTCTGAGCATTC | 96 |
| *HK1* | Hexokinase-1 | TTTCCCTTGTCGGCAATCCA | CCTCCACTCCGCTTGCTTTA | 80 |
| *HPRT1** | Hypoxanthine-guanine phosphoribosyltransferase | TATGGACAGGACTGAACGGC | ACACAGAGGGCTACGATGTG | 75 |
| *IFNG* | Interferon γ | AGCTTTGCGTGACTTTGTGT | ATGCTCCTTTGAATGGCCTG | 247 |
| *IL2* | Interleukin 2 | AAGCTCTGGAGGGAGTGCTA | CAACAGCAGTTACTGTCTCATCA | 159 |
| *IL4* | Interleukin 4 | GTACCAGCAACTTCGTCCAC | CCTTCTCCGTCGTGTTCTCT | 150 |
| *IL6* | Interleukin 6 | TGCCACCTCAGACAAAATGC | AGGTTCAGGTTGTTTTCTGCC | 159 |
| *IL10* | Interleukin 10 | GTCCGACTCAACGAAGAAGG | GCCAGGAAGATCAGGCAATA | 73 |
| *IL12A* | Interleukin 12 | TCCTGGGAAAGTCCTGTCGT | GGTGAGGTCGCTAGTTTGGA | 81 |
| *IL17A* | Interleukin 17 | GCACACGGGCTGCATCAACG | TGCAACCAACAGTGACCCGCA | 149 |
| *MPO* | Myeloperoxidase | CCCGAGTTGCTTTCCTCACT | AAGAAGGGGATGCAGTCACG | 127 |
| *PDHA1* | Pyruvate dehydrogenase E1 α | GTCAGGAAGCTTGTTGCGTG | GGTAAAGCCATGAGCTCGGT | 86 |
| *PKM* | Pyruvate kinase | GCCCTGGACACTAAAGGACC | CAGCCACAGGACATTCTCGT | 147 |
| *PPARA* | Peroxisome proliferator-activated receptor alpha | CCGAGACCGCAGATCTCAAG | GACGAAAGGCGGGTTATTGC | 128 |
| *RORC* | RAR-related orphan receptor γ | CAGCGCTCCAACATCTTCTC | GACCAGCACCACTTCCATTG | 207 |
| *S100A9* | S100-A9 | GCCAAACTTTCTCAAGAAGCA | AGTGTCCAGGTCTTCCAGGAT | 70 |
| *TBX21* | T-box TBX21 (T-bet) | CTGAGAGTCGCGCTCAACAA | ACCCGGCCACAGTAAATGAC | 121 |
| *TGFB1* | Transforming growth factor beta-1 | GCAAGGTCCTGGCTCTGTA | TAGTACACGATGGGCAGTGG | 97 |
| *TLR2* | Toll-like receptor 2 | CGTGTGCTATGACGCTTTCG | GTACTTGCACCACTCGCTCT | 232 |
| *TLR4* | Toll-like receptor 4 | TGGTGTCCCAGCACTTCATA | CAACTTCTGCAGGACGATGA | 116 |
| *TNF* | Tumor necrosis factor α | ATTCAGGGATGTGTGGCCTG | CCAGATGTCCCAGGTTGCAT | 120 |
| Genes are ordered alphabetically according to gene symbol. AL, amplicon length. *, reference gene. | | | | |
